# Hydroxychloroquine and the associated risk of arrhythmias

**DOI:** 10.21542/gcsp.2024.17

**Published:** 2024-03-03

**Authors:** Hadi Farhat, Celine J. Kassab, Yehya Tlaiss, Sai Dheeraj Gutlapalli, Vijay Durga Pradeep Ganipineni, Jananthan Paramsothy, Sarah Tedesco, Tharunjan Kailayanathan, Razan Abdulaal, Philip Otterbeck

**Affiliations:** 1 Internal Medicine, University of Balamand, Beirut, Lebanon; 2 Pharmacy, Lebanese American University School of Pharmacy, Beirut, Lebanon; 3 Internal Medicine, Richmond University Medical Center Mount Sinai, Staten Island, New York, USA; 4 Internal Medicine, Thomas Hospital Infirmary Health, Fairhope, USA; 5 Psychiatry, Richmond University Medical Center Mount Sinai, Staten Island, New York, USA; 6 Cardiology, University of Balamand, Beirut, Lebanon

## Abstract

Hydroxychloroquine (HCQ), which was initially used as an antimalarial drug, is now being used to treat other illnesses, especially rheumatic autoimmune disorders  such as systemic lupus erythematosus, primary Sjögren’s syndrome, and rheumatoid arthritis, because it is safe, effective, and cost efficient. This drug has shown high efficacy and has become the first-line treatment for many of these diseases. Although HCQ has many therapeutic effects, it has unfortunately shown some complications, especially with its long-term use. One of these side effects is arrhythmia through prolongation of the QT interval. This narrative literature review focuses on the effects of HCQ on the QT interval in patients with rheumatologic diseases who have been prescribed this drug. In particular, we will focus on the increased risk of arrhythmia when HCQ is administered with other drugs, such as azithromycin and many others, along with drug-drug interactions. In addition, we investigated the safety of this drug in pregnant women.

## Introduction & background

Hydroxychloroquine (HCQ), a disease-modifying antirheumatic drug(DMARD), has been widely prescribed in patients with systemic lupus erythematosus (SLE) and rheumatoid arthritis (RA) for the treatment and prevention of malaria. HCQ is believed to cause arrhythmia by slowing the rate of action potential firing in the sinoatrial (SA) node. This could be explained by the structural similarity between HCQ and class IA anti-arrhythmic quinidine, which specifically blocks sodium and potassium channels, leading to prolongation of the QT interval and increasing the risk of Torsades de Pointes (TdP)^1,2,3,4,5^. However, conflicting data are available.

The QT interval, which is a measurement obtained from an electrocardiogram (ECG), can predict the development of arrhythmia. Typically, a normal QT interval lasts for approximately 400 ms (ms), with minor differences between men and women. If the corrected QT interval (QTc) exceeds 500 ms, it can result in a dangerous and possibly fatal heart rhythm disorder TdP^6,7,8,9^. Risk factors for QTc prolongation include being over 65 years old, having a slow heart rate, having an enlarged heart, being female, having low levels of magnesium or potassium, pre-existing heart disease, high levels of certain drugs in the bloodstream, reduced drug clearance due to kidney or liver failure, genetic variations in ion channels or cytochrome enzymes that cause slower drug metabolism, and congenital long QT syndrome^6,10,11,12^.

According to statistics provided by the Centers for Disease Control and Prevention (CDC), it is anticipated that the prevalence of doctor-diagnosed arthritis will increase in the upcoming decades^13^. The American College of Rheumatology Guidelines of 2021 recommend the use of DMARDs as first-line agents for the treatment of RA and SLE, and these guidelines are being implemented in many other countries, such as Saudi Arabia^14,15,16^.

Owing to its anti-inflammatory, immunomodulatory, and metabolic properties, HCQ has been increasingly used as the primary treatment for chronic conditions, such as SLE and RA. Both SLE and RA are debilitating diseases that mostly affect women of childbearing age. Such diseases need to be addressed to enhance quality of life, increase productivity, and minimize side effects. HCQ has regained attention, particularly because of its off-label emergency use in the treatment of COVID-19 in 2020.

In this narrative literature review, we focus on whether HCQ is associated with arrhythmias, such as QTc interval prolongation or more severe TdP. We specifically review these adverse effects when the drug is used in people with rheumatologic diseases, such as SLE and RA, as well as in pregnant women. We also analyzed whether there were any drug-drug interactions between HCQ and other drugs that might lead to a concomitant increased risk of QTc prolongation and possible TdP in patients with or without other comorbidities.

## Methods

The relevant data for our narrative literature review were gathered from the PubMed database. Nine keywords were used: “Hydroxychloroquine”, “Long QT Syndrome”, “Arrhythmia”, “Arthritis, Rheumatoid”, “Lupus Erythematosus, Systemic”, “Autoimmune Diseases”, “Torsade de Pointes”, “Drug interactions”, and “Pregnancy” and the search was performed using Medical Subject Heading (MeSH) Strategy. We carefully screened and included all relevant articles from inception until August 2023. All the data were sourced from PubMed.

### Risk of arrhythmias in systemic lupus erythematosus and rheumatoid arthritis

Given the rise of arthritis cases worldwide and the use of DMARDs as first-line treatment (even among pregnant women), it is important to examine the adverse effects related to HCQ, particularly arrhythmia, to weigh the risks and benefits of prescribing such medications^17,18,19^.

A retrospective case-control study was conducted in Taiwan in May 2021 to investigate the cardiac safety of HCQ among patients with SLE^20^. The data for this study were gathered from the Taiwan National Health Insurance (NHI) program. A sum of 2,499 individuals who were recently diagnosed with SLE were identified, with 81% of them being female. Of these, 251 patients were included in the new-onset arrhythmia group, with an average age of 50.4 years. However, 251 individuals were in the non-arrhythmia group with an average age of 49.1 years. Only patients who were newly diagnosed with SLE with a minimum of two outpatient clinic visits or one admission between 2000 and 2012 were included in the study. Patients diagnosed with arrhythmia before SLE diagnosis were excluded from the study. Sensitivity analysis minimized confounding variables that could otherwise have affected the results. In addition, simple matching was performed to obtain a ratio of 1:1 according to age, sex, and year of SLE diagnosis.

This study revealed that, in comparison to the non-HCQ group, the associated risk of arrhythmia did not increase with HCQ treatment, regardless of the duration of treatment <180 days or >180 days (adjusted ORs 1.61, and 1.44; *p*  = 0.133 and 0.122, respectively), <50% or >50% adherence rate (adjusted ORs 1.62 and 1.37, *p* = 0.084, and 0.235, respectively), or a daily dose of <400 mg or >400 mg (adjusted ORs 1.47 and 1.53; *p* = 0.096 and 0.210, respectively). With a 95% CI, the sensitivity analysis showed no correlation between comorbidities and the risk of arrhythmia. In conclusion, the findings showed that patients with SLE who took HCQ did not have an increased likelihood of experiencing any type of cardiac arrhythmia or conduction disturbance. Additionally, the chance of developing arrhythmia was not affected by the length of time HCQ was administered, a higher daily dosage, or improved adherence to the medication. One limitation of this study was the absence of ECG data from the Longitudinal Health Insurance Database, which prevented  the accurate assessment of QTc intervals. In addition, the investigation did not consider other drugs that could potentially prolong QT intervals, possibly affecting the findings of the study. In addition, a lack of access to serum blood test results (*e.g.*, potassium, calcium, magnesium, glucose, and thyroid function) may influence arrhythmia development. However, researchers believe that their results on arrhythmia risk in HCQ therapy are still reliable.

In a comprehensive cohort study conducted by Quiñones et al., 8,852 veterans newly diagnosed with RA were meticulously examined^21^. This rigorous investigation aimed to assess the safety profile of HCQ through a propensity score-matched active comparator study within this cohort. The demographic composition of the study participants was characterized by a mean age of 64 years with a standard deviation of 12 years, with 14% being women and 28% being of Black ethnicity. This sizable cohort was evenly divided, with 4,426 individuals receiving HCQ treatment and the remaining 4,426 individuals receiving other DMARDs, ensuring a well-balanced comparison across 87 key characteristics.

The occurrence of LQTS was closely monitored over the course of a 19-year follow-up period. In the initial two years of the study, LQTS was observed in only four patients (0.09%) within the HCQ group, while five patients (0.11%) from the other DMARDs group experienced this condition. However, as the study continued, these incidence rates evolved. In the subsequent five-year period, LQTS cases increased to 17 (0.38%) in the HCQ group and 6 (0.14%) in the other DMARDs group. Interestingly, in the subsequent five years, the HCQ group saw only five additional cases, with just one occurring over the following nine years.

In summary, the study’s authors arrived at a compelling conclusion: there was no discernible association between HCQ usage and LQTS during the initial two years of treatment. Nevertheless, a slightly elevated risk emerged during the subsequent five years of therapy. However, it is important to note that the absolute 5-year risk remained remarkably low, comprising only 0.67% of the entire cohort of 8,852 individuals with an absolute risk difference of 17 cases. Notably, both risks diminished with longer-term follow-up. Considering these findings, this study provides valuable evidence to support the long-term safety profile of HCQ in the context of RA treatment.

Turning our attention to a population-based study conducted by Hoque et al., the authors explored the potential connection between HCQ initiation and the risk of arrhythmia in patients newly diagnosed with either RA or SLE^22^. Their extensive investigation spanned the period from January 1996 to December 2014, encompassing a substantial cohort of patients. These individuals were categorized into two groups: the first group consisted of 11,518 patients who received HCQ prescriptions(referred to as HCQ initiators), while the second group comprised an equal number of 11,518 individuals who did not receive HCQ prescriptions (referred to as HCQ non-initiators) each year under study. The researchers diligently monitored the occurrence of new-onset arrhythmias, including atrial fibrillation, abnormal ECG encompassing LQTS and conduction disorders, as well as other unspecified arrhythmias during the follow-up period.

Throughout the comprehensive eight-year follow-up period, a total of 1,610 incident arrhythmias were documented in the HCQ initiator group, while a slightly higher count of 1,646 incident arrhythmias was observed in the HCQ non-initiator group. The crude incidence rates of arrhythmias were calculated to be 17.5 and 18.1 cases per 1,000 persons per year for the HCQ initiator and non-initiator groups, respectively. The analysis and findings of this study led the authors to conclude that the initiation of HCQ therapy did not yield an elevated risk of any type of arrhythmia among individuals new to HCQ therapy.

In a separate cohort study conducted by Nishiyama et al., the primary objective was to evaluate the impact of HCQ treatment on QTc interval among patients diagnosed with SLE during the period spanning from 2015 to 2020^23^. In addition to this primary goal, the study also aimed to identify various factors that could potentially influence QTc prolongation. Their research cohort comprised 126 patients with SLE, of whom 42 received HCQ treatment, while the remaining individuals served as the control group, receiving no HCQ treatment. QTc intervals were assessed using ECGs both before and after the administration of HCQ, and the results were compared to those of the control group. Within the HCQ-treated group, the mean QTc interval exhibited a significant increase (*p* < .001), whereas no statistically significant difference was observed in the mean QTc interval within the control group. However, upon conducting a comprehensive multiple logistic regression analysis, the researchers found no significant disparities between the two groups.

The pivotal conclusion drawn from this study suggests that HCQ administration could lead to QTc prolongation in patients with SLE. They also advise exercising caution when prescribing HCQ, particularly among patients with an extended duration of SLE, considering the potential for QTc prolongation as a relevant factor in their treatment.

Moreover, in another cohort study conducted by Costedoat-Chalumeau et al., an investigation was conducted involving 85 patients diagnosed with connective tissue diseases who were undergoing HCQ treatment^24^. To assess the potential risk of arrhythmias in these patients, researchers utilized 12-lead ECGs, tracking their cardiac health for a minimum duration of one year. Throughout this study, the investigators observed two cases of incomplete right bundle branch blocks and one case of a left bundle branch block. Importantly, no cases of atrioventricular block were detected. The collected data revealed a mean PR interval of 137 ± 20 ms, with a range spanning 99–188 ms. Similarly, the mean QTc interval was 410 ms, spanning a range of 349–464 ms. The mean heart rate was 73 beats/min, ranging from 53 to 102 beats/min.

**Figure 1.**
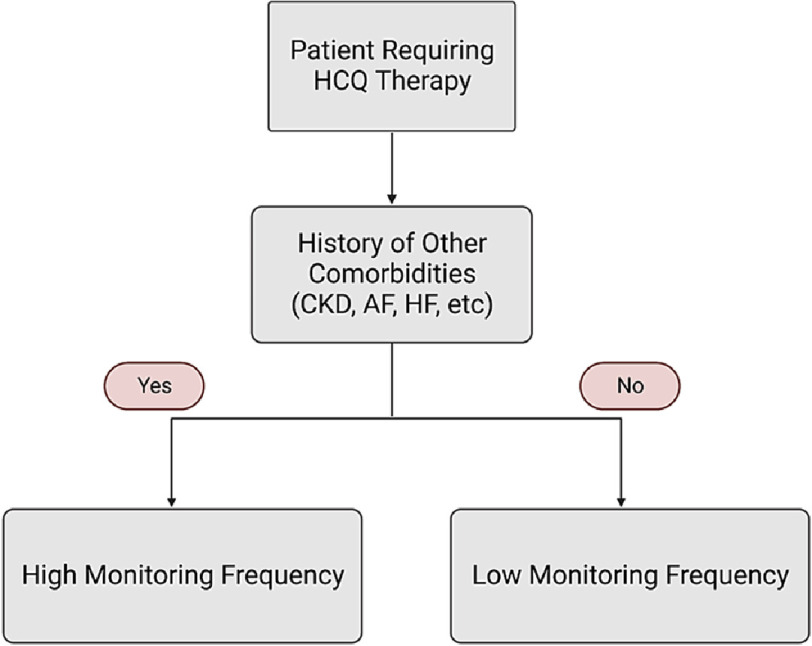
Algorithm for hydroxychloroquine monitoring for arrhythmias. Credits: Hadi Farhat and Sai Dheeraj Gutlapalli.

Remarkably, the researchers’ analysis indicated that both the PR interval and QTc interval fell within the range of normal values, while the heart rate remained consistent with typical ranges. Notably, the incidence of heart conduction disorders observed in this cohort closely mirrored the expected rates found in the general population. This compelling set of findings reinforces the existing body of evidence that supports the safety profile of HCQ.

In 2020, a study conducted in Minneapolis revealed that a considerable proportion of patients who take HCQ experience QT prolongation; this effect is even more likely to occur in patients with chronic kidney disease, atrial fibrillation, and/or heart failure (Figure 1). This retrospective study was conducted between 2000 and 2020 in 819 patients receiving HCQ for the treatment of rheumatologic conditions. The focus was on measuring the QTc interval using the Bazett formula. Nevertheless, this study had a major limitation: the results were based on patients with SLE who also had COVID-19, which increased the risk of cardiac conductivity abnormalities. This makes it difficult to determine whether the observed changes in cardiac conductivity were solely due to HCQ or whether COVID-19 also played a role. Therefore, the findings of this study should be interpreted with moderate caution^25^.

In addition to  cohort and population studies, numerous case reports within scientific literature have highlighted an elevated susceptibility to arrhythmias in individuals with autoimmune disorders. One such case report focused on a 41-year-old female patient with SLE and a history of cardiac and renal abnormalities^26^. Following HCQ administration, a noteworthy extension of the QTc interval was documented. Fortunately, the decision to discontinue HCQ ultimately led to the restoration of her QTc interval to normal levels over a span of one year, attributable to the prolonged half-life of the drug.

An additional case merits attention, involving a 67-year-old female afflicted with SLE^27^. The patient’s presentation was marked by LQTS, culminating in a diagnosis of TdP in the emergency room. After ruling out other plausible causes of LQTS, suspicion arose regarding HCQ as an instigator of ventricular tachycardia. Upon cessation of HCQ, a notable reduction in the QT interval was observed. Consequently, the authors underscored the necessity for a judicious balance between the chronic utilization of HCQ for rheumatic conditions or as an antimalarial agent and the potential peril of precipitating life-threatening cardiac arrhythmias.

Another noteworthy case comes from the work of Comin-Colet et al., which featured a 40-year-old woman grappling with SLE^28^. This patient experienced acute exacerbation of SLE and was subsequently prescribed long-term HCQ therapy. Remarkably, she developed a rare and concerning complication: complete heart block. While complete heart block can be a known complication of SLE, the authors assert that HCQ may elevate the risk of its manifestation.

### Safety of hydroxychloroquine with other medications such as azithromycin in patients with lupus

Another key point is to examine the safety of the concomitant use of HCQ and other medications. This is particularly important because polypharmacy is highly prevalent in patients with RA and SLE^29^.

Numerous investigations have delved into the potential drug-drug interactions (DDIs) arising from the combination of HCQ and azithromycin among patients afflicted with connective tissue diseases.

A comprehensive comparative analysis conducted by Sarayani et al. aimed to evaluate the potential risks associated with the use of HCQ/chloroquine (CQ) and azithromycin, specifically focusing on QT segment prolongation, TdP, and mortality among patients^30^. To gather data, researchers extracted information from the U.S. Food and Drug Administration’s Adverse Event Reporting System. The assessment encompassed various groups including those exposed to HCQ/CQ alone, azithromycin alone, HCQ/CQ + azithromycin, amoxicillin alone, and HCQ/CQ + amoxicillin alone. Amoxicillin served as the control group for comparison.

The findings revealed significant differences in Proportional Reporting Ratios (PRRs) and their corresponding 95% Confidence Intervals (CIs). Specifically, HCQ/CQ use alone showed a PRR of 1.43 (95% CI [1.29–2.59]), whereas azithromycin alone exhibited a notably higher PRR of 4.10 (95% CI [3.80–4.42]) in relation to TdP/QT prolongation. In the combined group of HCQ/CQ + azithromycin, the PRR and 95% CI were 3.77 (1.80−7.87). Ultimately, the study’s conclusion emphasized that the utilization of HCQ/CQ alone appeared to be safe, whereas the use of azithromycin alone or in conjunction with HCQ/CQ was associated with a significant risk of QT prolongation. Notably, it is important to highlight that this research did not focus on COVID-19 patients; instead, it encompassed populations utilizing these medications for common conditions, such as SLE and sinus infections.

Saint-Gerons et al. conducted another insightful retrospective analysis, employing the extensive World Health Organization (WHO) database as their data source^2^. Their study focused on patients treated with HCQ, CQ, and azithromycin who reported instances of QT prolongation and TdP.

The incidence of TdP among these patients was as follows: CQ: 11 cases, HCQ: 31 cases, CQ + HCQ: 1 case, HCQ + azithromycin: 27 cases, and azithromycin: 100 cases. Notably, HCQ was primarily prescribed for conditions such as SLE, RA, and various other connective tissue diseases. COVID-19 was not a confounding factor in the present study.

The authors advocated for a cautious approach to prescribing CQ, HCQ, and azithromycin, emphasizing that these medications should be reserved for therapeutic indications with well-established positive benefit-to-risk ratios. Importantly, both healthcare providers and patients must be vigilant regarding the potential cardiotoxic effects associated with these drugs.

An experimental placebo-controlled double-blind randomized multicenter trial evaluated the efficacy of HCQ and azithromycin in COVID-19 patients. Patients enrolled in the study had to have a positive PCR test. The intervention was 500 mg daily of azithromycin for three days followed by 250 mg daily azithromycin for 12 days, combined with 200 mg twice-daily HCQ for all 15 days. The control group received a placebo. Baseline characteristics were balanced among the groups. During follow-up, one (1.64%) out of 61 patients in the HCQ plus azithromycin group and two (3.6%) out of 56 patients in the placebo group had a recorded QTc >500 ms^31^.

The authors’ conclusions point to generally neutral outcomes in patients who underwent treatments involving HCQ as compared to standard care, azithromycin as compared to standard care, or the combined administration of azithromycin and HCQ. Notably, a slight elevation in QTc values was observed among patients who were administered high-dose HCQ/CQ, whereas those who received the recommended standard doses of HCQ/CQ did not display prolonged QTc values. Importantly, it is worth acknowledging that the study cohort consisted of patients with SLE and the presence of COVID-19 introduced a confounding variable that may have influenced the study outcomes.

Another clinical trial was conducted in a hospital in São Paulo, Brazil to evaluate the effectiveness of HCQ plus azithromycin in SLE patients with mild COVID-19 infection. The trial was double-blind, randomized, and placebo-controlled. Results showed that on day nine, patients in the treatment arm had a longer QTc interval compared to the placebo group, with the difference being statistically significant (intention-to-treat: 406.5 ms *vs.* 398.3 ms, *p* = 0.024; per-protocol: 406.1 ms *vs.* 397.5 ms, *p* = 0.069). A key point is that these patients had a low cardiovascular risk; however, no patient had to stop taking the drug because of this event^32^.

In addition, a multicenter, randomized, open-label, three-group, controlled trial was published involving hospitalized patients with suspected or confirmed COVID-19 who were receiving either no supplemental oxygen or a maximum of 4 L/min of supplemental oxygen. Patients were randomly assigned to three groups in a 1:1:1 ratio to receive the following: standard care, standard care plus HCQ at a dose of 400 mg twice daily, or standard care plus HCQ at a dose of 400 mg twice daily plus azithromycin at a dose of 500 mg once daily for 7 days. This study revealed that concerns about QTc interval prolongation were directed more often towards patients in the intervention groups. Patients who were given HCQ in addition to azithromycin or HCQ alone had a higher incidence of QTc interval prolongation compared to the control group (HCQ plus azithromycin *vs.* control: odds ratio (OR), 0.99; 95% CI, 0.57 to 1.73; *p* = 1.00; HCQ alone *vs.* control: OR, 1.21; 95% CI, 0.69 to 2.11; *p* = 1.00; and HCQ plus azithromycin *vs.* HCQ alone: OR, 0.82; 95% CI, 0.47 to 1.43; *p* = 1.00). However, the increased risk was not statistically significant. Patients in the control group were not monitored as closely as those in the intervention arm^33^.

Finally, a retrospective cohort study using data from patients enrolled in the Beaumont Health COVID-19 Database of COVID-19 hospitalized patients treated with HCQ/azithromycin showed a significant increase in QTc among these patients. However, these events did not increase the risk of mortality. Factors associated with QTc prolongation >500 ms were age (*p* ¡ 0.001), body mass index <30 kg/m^2^ (*p*¡0.005), heart failure (*p*¡0.001), elevated creatinine (*p* < 0.005), and peak troponin (*p* < 0.001)^34^.

### Other drug-drug interactions associated with QT prolongation

**Figure 2.**
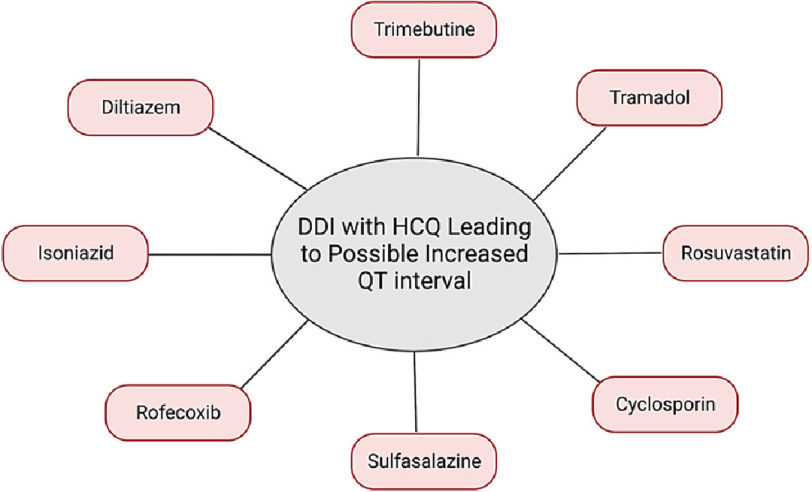
Increased risk of QT prolongation with the use of HCQ and other drugs. Credits: Hadi Farhat and Celine J. Kassab.

A major retrospective case-control study was conducted to evaluate the risk of QT prolongation, defined as exceeding 450 ms for males and 460 ms for females, caused by HCQ and other medications^35^. The data were collected from the electronic medical record database of Ajou University Hospital, a tertiary teaching hospital in Korea. This study employed real-world data to explore potential DDIs  between HCQ and 118 other drugs. The findings indicated that 12 of these drugs(trimebutine, tacrolimus, tramadol, rosuvastatin, cyclosporin, sulfasalazine, rofecoxib, diltiazem, piperacillin/tazobactam, isoniazid, clarithromycin, and furosemide) had statistically significant DDIs with HCQ, which increased the risk of QT prolongation. Surprisingly, for eight of these drugs (tramadol, trimebutine, rosuvastatin, cyclosporin, sulfasalazine, rofecoxib, diltiazem, and isoniazid), the risk of QT prolongation was not observed with the use of these drugs alone (Figure 2). However, DDIs with HCQ increased the risk in this direction. The OR of these eight drugs to interact with HCQ were the following: trimebutine (OR = 2.17, 95% CI = 1.33−3.53, *p* < 0.001), tramadol (OR = 1.70, 95% CI = 1.24−2.34, *p* < 0.001), rosuvastatin (OR = 2.80, 95% CI = 1.40−5.58, *p* < 0.001), cyclosporin (OR = 8.06, 95% CI = 3.05–21.29, *p* < 0.001), sulfasalazine (OR = 2.12, 95% CI = 1.19−3.80, *p* = 0.01), rofecoxib (OR = 8.89, 95% CI = 1.56–50.7, *p* = 0.01), isoniazid(OR = 3.89, 95% CI = 1.17–12.98, *p* = 0.03), diltiazem (OR = 3.15, 95% CI = 1.29−7.72, *p* = 0.01).

Another study included 76 subjects with chest tomography results suggesting COVID-19 pneumonia who were administered a combination of HCQ and moxifloxacin (MOX) and underwent a standard 12-lead ECG on days two and five. The study measured the heart rate, QT interval, Tp-e interval, and Tp-e/QT ratio. Compared with day 2, the ECG results on day 5 exhibited notable changes, with a significant increase in several key parameters. Specifically, the QT interval showed a marked elevation (370.8 ± 32.5 *vs.* 381.0 ± 29.3, respectively, *p* = 0.001), as did the QTc interval (424 (403–436) *vs.* 442 (420–468), respectively, *p* < 0.001). Additionally, both the Tp-e interval (60 (55–70) *vs.* 65 (57–75), respectively, *p* < 0.001) and cTp-e interval(72.2 ± 12.9 *vs.* 75.4 ± 12.7, respectively, *p* < 0.001) demonstrated significant increases. Among the patient cohort, 5% exhibited a prolonged QTc interval exceeding 500 ms, whereas 8% experienced a notable QTc interval increase of more than 60 ms. Additionally, the Tp-e/QT ratio surpassed 0.23 in 4% of the patients. Notably, the therapy did not result in ventricular arrhythmia or TdP during the short-term observation period^36^.

Additionally, by examining health records from the Cerner Health Facts^®^ database, which contains data from over 60 million patients, the study found a link between HCQ and a moderate increase in QTc interval compared to those who took sulfasalazine or methotrexate^1^. When HCQ was used alone, the average QTc measurement before the index timestamp (a specific time point) was 428.15 ms. After the index timestamp was reached, it increased to 446.18 ms. However, when HCQ was used along with other medications known to prolong QTc intervals, the average QTc measurement before the index timestamp was 440.6 ms, and after the index timestamp, it was 441.0 ms. The authors concluded that there was no significant extra effect when HCQ was used with other drugs that could also increase the QTc interval.

Finally, in a documented case by Miranda-Aquino et al., a 67-year-old female diagnosed with SLE was prescribed a 200 mg dose of HCQ^37^. Given her pre-existing atrial fibrillation, her treatment plan included the addition of amiodarone. Two weeks after this combined treatment, ECG revealed an extended QT interval, a cardiac indicator. Subsequently, upon discontinuation of HCQ and amiodarone, the QT interval returned to its normal duration. Intriguingly, when amiodarone was cautiously reintroduced, the subsequent ECG showed a normalized QT interval. Thus, the authors concluded that prolonged QT syndrome manifested when amiodarone and HCQ interacted.

### Hydroxychloroquine in pregnancy

HCQ is a life-saving medication for patients with RA and SLE, particularly pregnant women. This is because it has lower teratogenic effects than other treatments such as methotrexate.

To further confirm its safety, a study revealed the cardiotoxic profile of HCQ in the neonatal/fetus^38^. The study collected neonatal ECGs and blood levels of HCQ while evaluating the effectiveness of 400 mg daily HCQ in preventing the recurrence of congenital heart block associated with anti-SSA/Ro antibodies. A total of 45 ECGs were available for assessing QTc. The HCQ levels were evaluated during each trimester of pregnancy and in cord blood, ensuring a clear understanding of drug exposure. The comprehensive analysis yielded reassuring results, as there was no discernible link between cord blood levels of HCQ and neonatal QTc prolongation (*R* = 0.02, *p* = 0.86), nor was there any significant association between the average HCQ values recorded across individual pregnancies and QTc (*R* = 0.04, *p* = 0.80). Notably, only five neonates, constituting 11%(with a 95% CI ranging from 4% to 24%), exhibited QTc prolongation exceeding two standard deviations above that of historical healthy controls, with two displaying marked prolongation and three showing marginal increases. It is worth mentioning that aside from these QTc variations, the ECGs were otherwise within the normal parameters. The authors concluded that these data offer reassuring evidence that maternal HCQ use is associated with a minimal occurrence of infant QTc prolongation.

It is important to note that this is the only study in the PubMed database that assesses the risk of QT prolongation in the fetuses of pregnant women with connective tissue diseases who take HCQ. Further research is required to address this issue. 

In contrast, much research has proven the therapeutic effects of HCQ in fetuses born with congenital heart block^39,40,41,42,43,44^. We will not address this question because our review focuses on the risk of arrhythmia in patients taking HCQ. 

### Discussion

This review provides a comprehensive overview of various studies and case reports investigating the risk of arrhythmias associated with the use of HCQ in the context of treating SLE and RA.

One of the key studies, a retrospective case-control study conducted in Taiwan involving patients with SLE, did not identify an increased risk of arrhythmia linked to HCQ treatment^20^. Regardless of factors such as treatment duration, adherence rate, or daily dosage, the findings from this study indicate that patients with SLE using HCQ were not at a higher likelihood of experiencing any type of cardiac arrhythmia or conduction disturbance. In addition to SLE, in a cohort study focusing on RA patients, there was no apparent association between HCQ usage and LQTS within the first two years of treatment^21^. Although a slightly elevated risk emerged during the subsequent five years of therapy, it is crucial to note that the absolute 5-year risk remained low, constituting only 0.67% of the entire cohort.

This conclusion was also confirmed in a population-based study conducted by Hoque et al., who explored the potential connection between HCQ initiation and the risk of arrhythmia in patients newly diagnosed with either RA or SLE^22^. This study found that the initiation of HCQ therapy did not result in a heightened risk of any type of arrhythmia among individuals new to HCQ therapy.

Furthermore, the impact of HCQ treatment on QTc interval in patients with SLE was investigated in another cohort study^23^. Although the study indicated a significant increase in the mean QTc interval in the HCQ-treated group, performing a comprehensive multiple logistic regression analysis did not reveal significant disparities between the HCQ-treated and control groups. This suggests that while HCQ might lead to QTc prolongation in some patients with SLE, the overall risk remains uncertain.

Additionally, a study conducted by Costedoat-Chalumeau et al. involving patients with connective tissue diseases receiving HCQ treatment found that the PR and QTc interval values fell within the range of normal values, further supporting the safety profile of HCQ^24^.

However, it is essential to acknowledge the study conducted in Minneapolis that reported QT prolongation in patients taking HCQ, particularly in those with chronic kidney disease, atrial fibrillation, and/or heart failure^25^. Nevertheless, since COVID-19 was a confounding variable in this study, it is challenging to determine whether the observed changes in cardiac conductivity were solely due to HCQ or whether COVID-19 also played a role.

Lastly, the case reports that we discussed have highlighted instances of QTc interval prolongation and arrhythmias in SLE patients following HCQ administration^26,27,28^. Importantly, these patients often had multiple comorbidities, which could have contributed to these findings. These cases emphasize the significance of monitoring cardiac health and considering the potential risk of arrhythmias when prescribing HCQ, particularly in patients with underlying cardiac issues.

We can conclude that most of the studies mentioned here confirmed the safety of HCQ in patients with connective tissue diseases, such as SLE and RA. However, when other comorbidities are present, the cause of arrhythmias, such as QT prolongation, is more challenging to diagnose.

The safety of HCQ when used concurrently with other medications such as azithromycin in patients with conditions such as SLE has garnered significant attention owing to the prevalence of polypharmacy in individuals with RA and SLE^29^.

A comprehensive analysis by Sarayani et al. highlighted the safety of HCQ/CQ alone but cautioned against the use of azithromycin alone or in combination with HCQ/CQ due to QT prolongation. Notably, this research did not involve COVID-19 patients but focused on common conditions such as SLE and sinus infections. Another study by Saint-Gerons et al., which conducted a retrospective analysis using the WHO database, focused on HCQ, CQ, and azithromycin in patients with instances of QT prolongation and TdP^2^. Their study also included patients without COVID-19. They emphasized the need for cautious prescription of these medications, particularly in patients with well-established benefit-to-risk ratios, underlining the importance of monitoring potential cardiotoxic effects. It is important to note that these two studies had no confounders, such as COVID-19, and only included patients with connective tissue diseases.

However, the four other included studies had COVID-19 as a confounder, which might have altered the results of the study. For instance, an experimental placebo-controlled study evaluating HCQ and azithromycin in SLE patients with COVID-19 revealed generally neutral outcomes, with a slight elevation in QTc values noted in high-dose HCQ/CQ recipients, which was not statistically significant^31^. This was also confirmed by a multicenter trial in COVID-19 patients, which found that concerns about QTc interval prolongation were directed more often towards the intervention groups receiving HCQ and azithromycin, but the increased risk was not significant^33^. These two studies showed no significant increase in QT interval prolongation.

Moreover, the trial in São Paulo, Brazil, assessed HCQ plus azithromycin in SLE patients with mild COVID-19 infection and found a statistically significant increase in the QTc interval in the treatment group, although patients did not discontinue treatment due to this effect^32^. A similar conclusion was found in the retrospective cohort study using data from the Beaumont Health COVID-19 Database which noted significant QTc prolongation with HCQ/azithromycin treatment but did not observe an increased risk of death, highlighting factors associated with QTc prolongation^34^.

Thus, based on these four studies, two found a significant increase in QT prolongation in patients with connective tissue diseases taking HCQ/AZM. However, the other two showed no significant increase.

The risk of QT prolongation associated with HCQ in combination with other medications is clarified by other findings that we focused on in this narrative review. To assess the potential DDIs of HCQ with different medications as well as its effect on QTc interval prolongation, several significant studies were analyzed.

One important study examined the possibility of QT prolongation caused by HCQ and 118 other medications using real-world data from a tertiary teaching hospital in Korea. Twelve medications were found to have statistically significant DDIs with HCQ, which increased the risk of QT prolongation^35^. Surprisingly, the risk of QT prolongation was not seen when eight of these medications were taken alone. This emphasizes the importance of considering potential DDIs when prescribing HCQ in conjunction with these particular medicines because such combinations may have a significant impact on the QTc interval.

Furthermore, the study focusing on COVID-19 pneumonia patients who received a combination of HCQ and MOX, reported elevations in the QTc interval, Tp-e interval, and Tp-e/QT ratio^36^. Although no ventricular arrhythmias were observed during the short-term observation period, these findings suggest that the combination of HCQ and MOX may potentially lead to QT prolongation. Therefore, close cardiac monitoring is crucial when using this drug combination, particularly in patients at a higher risk of arrhythmias.

Additionally, a comprehensive analysis of de-identified patient health records indicated that HCQ was associated with a moderate increase in the QTc interval compared to individuals exposed to sulfasalazine or methotrexate^1^. However, no further enhancement of the QTc interval was observed when HCQ was used concomitantly with other drugs known to increase the QTc interval. These results suggest that while HCQ may cause QTc prolongation, its risk may not be substantially amplified by other QT-prolonging medications.

Finally, in the clinical case, Miranda-Aquino et al. reported the potential risk of HCQ-induced QT prolongation when used in conjunction with amiodarone^37^. This case involved a patient with SLE who experienced an extended QT interval after receiving both HCQ and amiodarone. Notably, discontinuation of both drugs led to QT interval normalization, and cautious reintroduction of amiodarone resulted in similar outcomes ^37^. This case illustrates the complex and potentially reversible nature of DDIs involving HCQ, and underscores the importance of careful monitoring and management in clinical practice.

Regarding our third topic, the results presented in this study contribute valuable insights into the safety profile of HCQ use during pregnancy, particularly in the context of managing connective tissue diseases such as RA and SLE. HCQ has long been regarded as a crucial medication for pregnant women with these conditions because of its relatively lower teratogenic effects compared to alternative treatments such as methotrexate.

To further confirm the safety of HCQ during pregnancy, a comprehensive study evaluated its cardiotoxic effects on the neonatal/foetal population^38^. The study approach included monitoring HCQ levels across trimesters and in cord blood, ensuring a thorough understanding of drug exposure throughout pregnancy.

The study’s findings offered reassurance, as there was no discernible correlation between cord blood HCQ levels and neonatal QTc prolongation. Similarly, there was no significant association between the average HCQ values recorded across individual pregnancies and QTc intervals. The authors concluded that maternal HCQ use was associated with a minimal occurrence of infant QTc prolongation.

However, it is crucial to acknowledge that this study represents the only available investigation within the PubMed database that assesses the risk of QT prolongation in the fetuses of pregnant women with connective tissue diseases who take HCQ. Given the potential implications for maternal and fetal health, this paucity of research highlights the need for further investigation to comprehensively address this critical issue and provide a more robust evidence base.

In contrast, existing research has predominantly focused on the therapeutic benefits of HCQ in fetuses born with congenital heart block^39,40,41,42,43,44^. While these studies provide valuable insights into treatment, it is essential to recognize that the current review primarily focuses on the risks associated with arrhythmia in patients taking HCQ. This underscores the importance of a balanced perspective, considering both the therapeutic and potential adverse effects of HCQ, particularly in the context of pregnancy, to guide clinical decision making and ensure the best outcomes for both mothers and their infants.

### Limitations

When interpreting the results, it is important to consider the limitations of the narrative literature review. The sole reliance on articles found in the PubMed database, which may introduce bias, is one of the main limitations. This method may have overlooked pertinent studies that were either not indexed in PubMed or were published in other databases, which could have produced inaccurate or incomplete results. 

Another drawback is the absence of explicit mention of non-English articles. The review might have missed important data and studies from other regions by excluding studies written in languages other than English, which could have limited the generalizability of the results.

In our search for DDI between HCQ and azithromycin, some of the articles had COVID-19 as a confounder, which could have affected the results of the studies. Moreover, there is a clear literature gap regarding the risk of fetal arrhythmia when HCQ is administered during pregnancy.

The lack of a systematic review methodology, such as the Preferred Reporting Items for Systematic Reviews and Meta-Analyses (PRISMA) guidelines, may have led to bias during the selection of studies and data extraction for the review. The thoroughness and rigor of the review may have resulted from the absence of a systematic approach.

## Conclusions

In conclusion, the available literature regarding HCQ safety remains controversial. While many studies have advocated the safety of HCQ regarding its risk of arrhythmia, many other studies have advised caution when prescribing it, especially if other comorbidities and concomitant drug use are present. In general, it is safe for people with connective tissue diseases, such as SLE and RA; however, close monitoring is often advised when the drug is used with other QT prolonging agents. Additionally, more studies are needed to assess the utility of this drug in pregnant patients and its effects on the fetus. Meanwhile, it is up to the healthcare team to weigh the benefits and risks of prescribing this medication or exploring alternative options.

## Author statement

### Conceptualization

 Hadi Farhat, Celine J. Kassab, Sai Dheeraj Gutlapalli, Razan Abdulaal, and Philip Otterbeck.

### Data curation

 Vijay Durga Pradeep Ganipineni, Jananthan Paramsothy, and Tharunjan Kailayanathan.

### Formal analysis

 Vijay Durga Pradeep Ganipineni, Jananthan Paramsothy, and Tharunjan Kailayanathan.

### Investigation

 Hadi Farhat, Celine J. Kassab, and Sai Dheeraj Gutlapalli.

### Methodology

 Yehya Tlaiss and Sarah Tedesco.

### Project administration

 Yehya Tlaiss.

### Supervision

 Razan Abdulaal and Philip Otterbeck.

### Validation

 Razan Abdulaal and Philip Otterbeck.

### Visualization

 Razan Abdulaal and Philip Otterbeck.

### Writing - original draft

 Hadi Farhat, Celine J. Kassab, Yehya Tlaiss, Jananthan Paramsothy, and Sarah Tedesco.

### Writing - review & editing

 Yehya Tlaiss, Vijay Durga Pradeep Ganipineni, Sarah Tedesco, and Tharunjan Kailayanathan.
